# Identification of Serum Biomarkers to Monitor Therapeutic Response in Intestinal-Type Gastric Cancer

**DOI:** 10.3390/ijms25063129

**Published:** 2024-03-08

**Authors:** Laura F. Dagley, Jumana Yousef, Adele Preaudet, Andrea Loving, Andrew I. Webb, Matthias Ernst, Tracy L. Putoczki

**Affiliations:** 1 The Walter and Eliza Hall Institute of Medical Research, Melbourne, VIC 3052, Australia; 2 Department of Medical Biology, The University of Melbourne, Melbourne, VIC 3052, Australia; 3 Ludwig Institute of Cancer Research, Melbourne, VIC 3052, Australia; 4 The Olivia Newton John Cancer Research Institute, Melbourne, VIC 3084, Australia; 5 School of Cancer Medicine, La Trobe University, Melbourne, VIC 3084, Australia; 6 Department of Surgery, The University of Melbourne, Melbourne, VIC 3052, Australia

**Keywords:** biomarker, Interleukin-11, intestinal-type gastric cancer, mouse model, proteomics

## Abstract

There are a limited number of clinically useful serum biomarkers to predict tumor onset or treatment response in gastric cancer (GC). For this reason, we explored the serum proteome of the *gp130*^Y757F^ murine model of intestinal-type gastric cancer (IGC). We identified 30 proteins with significantly elevated expression in early *gp130*^Y757F^ IGC and 12 proteins that were significantly elevated in late *gp130*^Y757F^ IGC compared to age- and gender-matched wild-type mice. Within these signatures, there was an overlap of 10 proteins commonly elevated in both early- and late-stage disease. These results highlight the potential to identify serum biomarkers of disease stage. Since IGC in the *gp130*^Y757F^ model can be reversed following therapeutic inhibition of Interleukin (IL)-11, we explored whether the protein signatures we identified could be used to monitor tumor regression. We compared two different therapeutic modalities and found 5 proteins to be uniquely differentially expressed between control animals and animals halfway through treatment, with 10 differentially expressed at the end of treatment. Our findings highlight the potential to identify reliable biomarkers to track IGC tumor regression in response to treatment.

## 1. Introduction

Gastric cancer (GC) is the third most common cause of cancer-related deaths world-wide, with a higher incidence in the southern hemisphere [1,2]. Many GC patients are diagnosed at a late stage of the disease, when treatments are limited to surgical resection and chemotherapy with palliative intent [3,4]. The identification of reliable serum biomarkers would greatly aid early disease detection and inform treatment decisions. Indeed, serum-based GC biomarkers are utilized in routine clinical practice with the aim of early detection, disease monitoring, and improved treatment decisions; however, they have limitations [5,6]. These serum biomarkers include carcinoembryonic antigen (CEA) [7], which is increased in the serum of gastric adenocarcinoma patients, but not during the early stages of carcinogenesis, and thus is not a useful marker of early disease [8]. Additional prognostic serum biomarkers include CA19-9 [9] and CA72-4 [10], which are widely used in clinical practice; however, their sensitivity and thus efficacy for screening early GC is limited [5]. Thus, there remains an urgent need for the discovery of reliable biomarkers.

The *gp130*^Y757F^ murine model of intestinal-type gastric cancer (IGC) [11] has been widely utilized to understand the pathogenesis of IGC and to identify and test new treatment opportunities [12]. In this model, the membrane proximal tyrosine (Y) residue on the cytoplasmic tail of the transmembrane glycoprotein 130 (gp130) receptor has a phenylalanine knock-in substitution (Y757F), resulting in hyper-activation of the signal transducer and activator of transcription (STAT3) protein in response to Interleukin (IL)-6 family cytokines. The *gp130*^Y757F^ mice develop tumors in the antrum of the stomach that, like human IGC, have elevated IL-6 family cytokine and STAT3 activation, which are associated with poor prognosis in human IGC [13,14]. In particular, IL-11 has been shown to be critical to disease onset and progression in the *gp130*^Y757F^ IGC model [15] and can be therapeutically manipulated to prevent and reverse tumor progression [15]. Previous studies utilizing this model identified four serum biomarkers, namely afamin, clusterin, vitamin D-binding protein (VDBP), and haptoglobin (Hb), as accurate indicators of the presence of IGC in both the *gp130*^Y757F^ model and in patients, that were reported to be superior to CA72-4 [16,17].

We employed the *gp130*^Y757F^ model of IGC, as a surrogate of human disease, for the identification of potential new biomarkers of disease progression and treatment response. We performed an unbiased label-free quantitative proteomics analysis of serum collected from *gp130*^Y757F^ mice to determine if a panel of biomarkers could distinguish early from late IGC. Given the demonstrated therapeutic benefit of IL-11 signaling inhibition in this model [15], we also sought to determine if the serum protein signatures that correlated with tumor progression were altered following therapeutic manipulation of tumor burden. Given the emerging interest in biomarkers that can predict treatment response in routine clinical practice, our results identified several potential serum protein biomarkers that may inform clinical trial design.

## 2. Results

### 2.1. gp130^Y757F^ Mice Have a Distinct Serum Proteome

**Figure 1:**
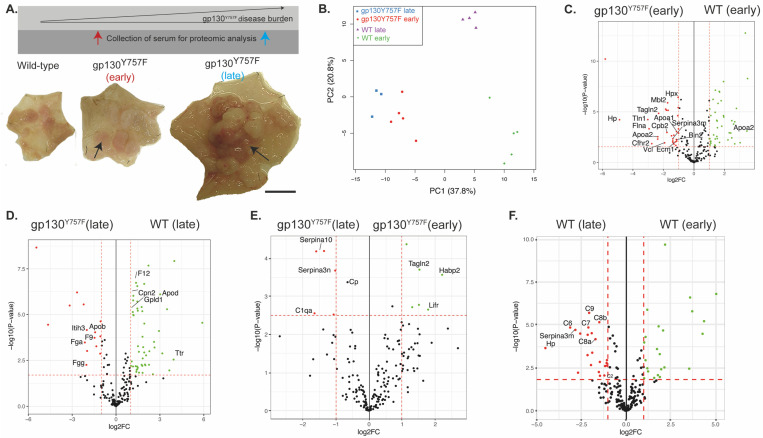
Identification of early- or late-stage GC serum biomarkers. (**A**) *gp130*^Y757F^ mice with early GC (2 months of age, N = 5 mice per cohort) and *gp130*^Y757F^ mice with late GC (6 months of age, N = 3–5 mice per cohort). Scale bar = 6mm. Red arrow = early collection time-point, blue arrow = late collection time-point, black arrows = macroscopic tumor; (**B**) principal component analysis (PCA) plot of samples colored by different groups illustrating a separation of the samples based on the first principal components; volcano plots illustrating the log_2_ protein ratios comparing (**C**) wild-type versus *gp130*^Y757F^ mice with early GC; (**D**) wild-type versus *gp130*^Y757F^ mice with late GC; (**E**) *gp130*^Y757F^ mice (early versus late GC); (**F**) wild-type mice (early versus late). Proteins were deemed differentially regulated if the log2 fold change in protein expression was ≥1-fold and exhibited an adjusted *p*-value ≤ 0.05.

We employed a label-free quantitative proteomics approach to determine if we could identify serum biomarkers that could be correlated with disease stage in the *gp130*^Y757F^ model. To this end, serum from terminal cardiac bleeds was collected from 2-month-old *gp130*^Y757F^ mice with early gastric disease for comparison with age-matched wild-type mice, as well as from 6-month-old *gp130*^Y757F^ mice with established disease for comparison with age-matched wild-type mice (Figure 1A). Principle component analysis (PCA) revealed a clear separation between the 2-month-old and 6-month-old wild-type mice, as well as between the wild-type and *gp130*^Y757F^ mice (Figure 1B). We also identified 18 proteins uniquely differentially expressed between the 2-month-old wild-type and *gp130*^Y757F^ mice (Figure 1C and Figure S1), 21 uniquely differentially expressed proteins between the 6-month-old wild-type and *gp130*^Y757F^ mice (Figure 1D and Figure S1), and only 2 uniquely differentially expressed proteins between the early- and late-stage disease in *gp130*^Y757F^ mice (Figure 1E and Figure S1).

**Figure 2:**
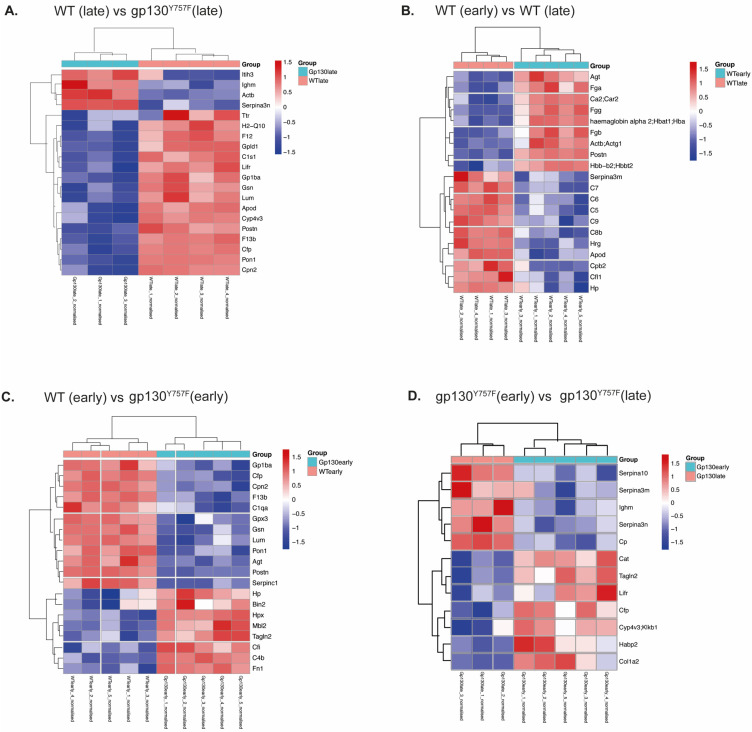
Heatmaps displaying the log intensities of the most differentially expressed proteins across the pairwise comparisons. (**A**) Wild-type versus *gp130*^Y757F^ mice with late GC; (**B**) wild-type (early versus late); (**C**) wild-type (early) versus *gp130*^Y757F^ mice with early GC; (**D**) *gp130*^Y757F^ mice (early versus late GC).

Pairwise comparison revealed that Hp, transgelin-2 (Tagln2), bridging integrator 2 (Bin2) and mannose-binding protein C (Mbl2) were significantly elevated in the serum of 2-month-old *gp130*^Y757F^ mice compared to wild-type mice and these differences were exclusive to the early GC disease stage (Figure 1C). In the 6-month-old *gp130*^Y757F^ mice with established disease, inter-alpha-trypsin inhibitor heavy chain H3 (Itih3) exhibited an increase in protein expression, while coagulation factor XII (F12) and transthyretin (Ttr) exhibited decreased protein expression compared to age-matched wild-type mice, which was unique to the late-disease stage (Figure 1D and Figure 2A).

Comparison between the early and late disease in *gp130*^Y757F^ mice revealed an increase in ceruloplasmin (Cp), protein Z-dependent protease inhibitor (Serpina10), and serine protease inhibitor A3N (Serpina3n) in late disease, suggesting that these proteins are unique indicators of disease stage (Figure 1E). Importantly, these proteins did not exhibit significant protein expression differences in the wild-type mice when comparing early to late samples (Figure 1F). Within the comparison between 2- and 6-month-old wild-type mice, we observed an increase in the expression of 22 proteins in the 6-month-old wild-type mice, including Hp and several complement components (C8, C9) previously associated with ageing in mice [18,19] (Figure 1F). These results suggest that previous observations linking haptoglobin to IGC may be associated with age, rather than a direct reflection of disease burden [16,17].

In contrast to previous studies [16], we did not observe an increase in afamin, clusterin, VDBP, or A1AT in the serum of tumor-bearing *gp130*^Y757F^ mice versus wild-type in either early- or late-stage diseases (Figure 2A–D). However, we did find that Tagln2, Habp2, and Lifr were elevated in early disease in *gp130*^Y757F^, while C1qa, Serpina3n, and Serpina10 were elevated in late disease (Figure 1E and Figure 2D), indicating that they may re-present biomarkers of disease, further supported by the fact that these proteins did not change in the same comparison in wild-type mice (Figure 1F).

### 2.2. Therapeutic Modulation of IL-11 Signaling Alters the Serum Proteome

**Figure 3:**
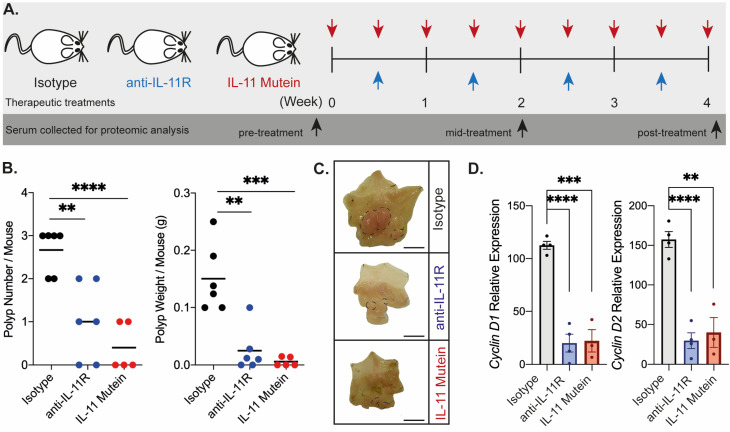
Therapeutic inhibition of IL-11 signaling. (**A**) *gp130*^Y757F^ mice with established gastric tumors were treated with an isotype control (black), or anti-IL11R antibody (blue), or IL-11 Mutein-PEG (red) for 4 consecutive weeks. (**B**) Total gastric tumor number and mass for the treatment group is indicated. Horizontal lines indicate the mean. **** *p* < 0.0001, *** *p* < 0.001, ** *p* < 0.01 Student’s *t*-test. (**C**) Representative whole mounts of stomachs from each treatment group. Scale bar = 6mm. Dotted lines outline regions of macroscopic tumor. (**D**) Relative gene expression for isolated gastric epithelial cells from each treatment group presented +/− SEM. **** *p* < 0.0001, *** *p* < 0.001, ** *p* < 0.01 Student’s *t*-test.

Since the gastric tumors that form in *gp130*^Y757F^ mice are exquisitely sensitive to the inhibition of IL-11 signaling [15], we systemically treated age-matched mice with an anti-IL-11R monoclonal antibody (CSL-4E5) [20] or IL-11 Mutein-PEG [15] (a recombinant protein that is a potent IL-11 signaling antagonist; Figure 3A). We found that either modality of IL-11 signaling inhibition significantly reduced overall tumor burden (Figure 3B,C). The reduction in tumor burden observed was attributed to reduced epithelial proliferation (Figure 3D and Figure S2). IL-11 is involved in megakaryocyte maturation and erythropoiesis [21]. We observed no adverse effect on platelet number (Figure S3), similar to our previous reports for therapeutic inhibition of IL-11 signaling [15] indicating a favorable safety profile.

**Figure 4:**
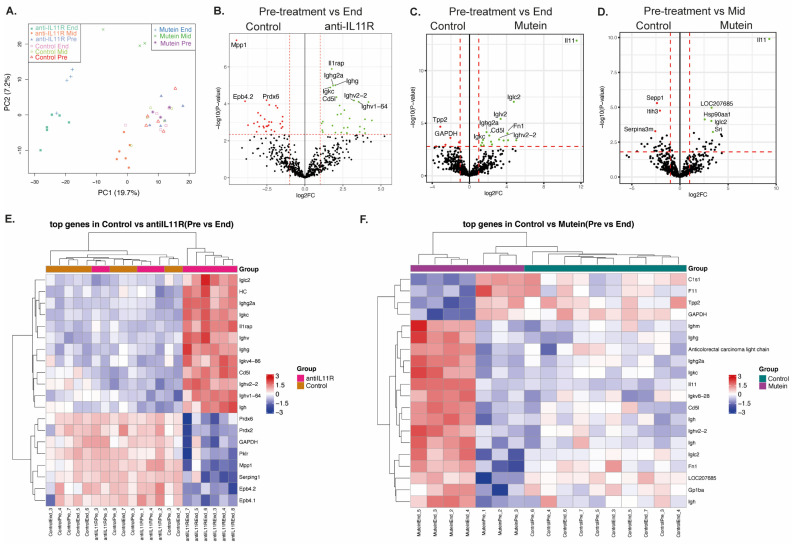
Serum biomarkers following therapeutic inhibition of IL-11 signaling. (**A**) Principal component analysis (PCA) plot of samples colored by different groups illustrating a separation of the samples based on the first principal components. Volcano plots illustrating the log2 protein ratios comparing pre-treatment versus end of treatment between (**B**) control-treated mice and anti-IL-11R mAb-treated mice and (**C**) IL-11 Mutein-treated mice and (**D**) pre-treatment versus mid-treatment IL-11 Mutein-treated mice. Proteins were deemed differentially regulated when the log2 fold change in protein expression was ≥1-fold and exhibited an adjusted *p*-value ≤ 0.05. Heatmaps displaying the log intensities of the most differentially expressed proteins across the pairwise comparisons, including pre-treatment versus end of treatment between (**E**) control-treated mice and anti-IL-11R mAb-treated mice and (**F**) Mutein-treated mice.

For each mouse, the serum that was collected prior to, during (mid-treatment protocol), and at the completion of the treatment protocol (Figure 3A) underwent label-free quantitative proteomics analysis to determine if a protein signature that correlated with treatment response could be identified. PCA plots demonstrated that the serum collected prior to treatment clustered together for both treatment groups, while the end-treatment IL-11 Mutein and anti-IL11R mAb groups also clustered together (Figure 4A). We then performed pairwise analyses to compare the differences in protein expression between the pre-treatment (Pre) versus halfway through the treatment (Mid), and pre-treatment versus the completion of the treatment (End), for both the anti-IL-11R mAb- and IL-11 Mutein-treated groups compared with the control-treated *gp130*^Y757F^ mice. In the serum collected prior to treatment versus at the end of the experiment, there were 48 unique proteins differentially expressed in the serum of mice treated with anti-IL-11R mAb when compared to the control animals and 7 unique proteins that were differentially expressed in the IL-11 Mutein-treated mice when compared to the control animals (Figure S4). Within these proteins, 10 proteins overlapped between both treatment groups, including several immunoglobulins (Igh, Ighm, Igkc) and CD5 antigen-like protein (Cd5l), which exhibited increased expression in the treated mice and may be indicators of treatment success (Figure 4B,C).

We found that at the completion of therapeutic treatment with the anti-IL11R mAb, several proteins, including membrane palmitylated protein 1 (Mpp1), peroxiredoxin 6 (Prdx6), and erythrocyte membrane protein band 4.2 (Epb4.2), were significantly increased in the control-treated mice, and interleukin-1 receptor accessory protein (Il1rap), Cd5l, and several immunoglobulins were decreased (Figure 4B), suggesting that these may be markers of treatment success. At the completion of the IL-11 Mutein treatment, a significant increase in IL-11 protein was detectable in the IL-11 Mutein-treated mice compared with control-treated mice, corresponding with the recombinant IL-11 Mutein provided for treatment (Figure 4C). We also saw increased expression of coagulation factor XI (F11), tripeptidyl peptidase 2 (Tpp2), and complement component 1 (C1s1) in the control-treated mice, suggesting that their decrease in the *gp130*^Y757F^ mice was an indicator of treatment success (Figure 4C). In addition, there was an increase in Fn1, Cd5l, and platelet glycoprotein 1b (Gp1ba) in the IL-11 Mutein-treated group (Figure 4C).

We found that within the serum collected prior to treatment versus halfway through the treatment protocol, there were no significant differentially expressed proteins within the mice treated with the anti-IL-11R mAb compared with control-treated mice (Figure S4). We did, however, observe a significant increase in the expression of proteins in the end-treatment anti-IL-11R mAb mice compared to the same mice pre-treatment, including Mpp1, Ephb4.1, and Ephb4.2 (Figure S5A). We also observed decreased expression of several immunoglobulins which was maintained at the end of the treatment (Figure S5A). Compared to the control-treated mice, there were five unique differentially expressed proteins in the IL-11 Mutein mid-treatment group (Figure S4), including Sepp1, Itih3, and Serpina3m, which were elevated in the control-treated mice compared with the Mutein-treated mice, suggesting that they may indicate treatment success (Figure 4D). Itih3 was previously found to be increased in late GC disease in *gp130*^Y757F^ mice compared to wild-type mice (Figure 1D). We also observed a significant increase in the expression of Itih3, Glo1, Anxa7, and Col1a1 in the pre-treatment Mutein mice compared to the same mice mid-treatment (Figure S5B). This further supports Itih3 as an early indicator of treatment response.

In addition, we observed increased expression of Il-11, Cd5l, Gp1ba, and several immunoglobulins at the IL-11 Mutein treatment endpoint, compared to the same mice pre-treatment (Figure S5B). The increase in IL-11 corresponds to the IL-11 Mutein-PEG in circulation, while immunoglobulins are associated with stromal remodeling in early gastric cancer [22]. Together with the observations in the anti-IL-11R antibody groups, these results further suggest that alterations in the tumor matrix are detectable in the serum proteome and reflect treatment response.

## 3. Discussion

GC is a devastating disease for which there are few treatment options [3]. Animal models have greatly increased our understanding of the pathophysiology of the disease and enabled the study of potential new therapeutic opportunities [23]. However, missing from the pipeline is the co-discovery of biomarkers to identify early disease or biomarkers to indicate that a treatment is impacting tumor growth. Here, we have utilized an animal model of IGC to identify potential blood-based biomarkers unique to disease stage, in addition to biomarkers that may predict tumor regression, with a focus on therapeutic inhibition of IL-11 signaling. These studies are timely, as monoclonal antibodies targeting IL-11 or its receptor have entered Phase I/II clinical trials [24].

### 3.1. Serum Biomarkers of Advanced Disease

**Figure 5:**
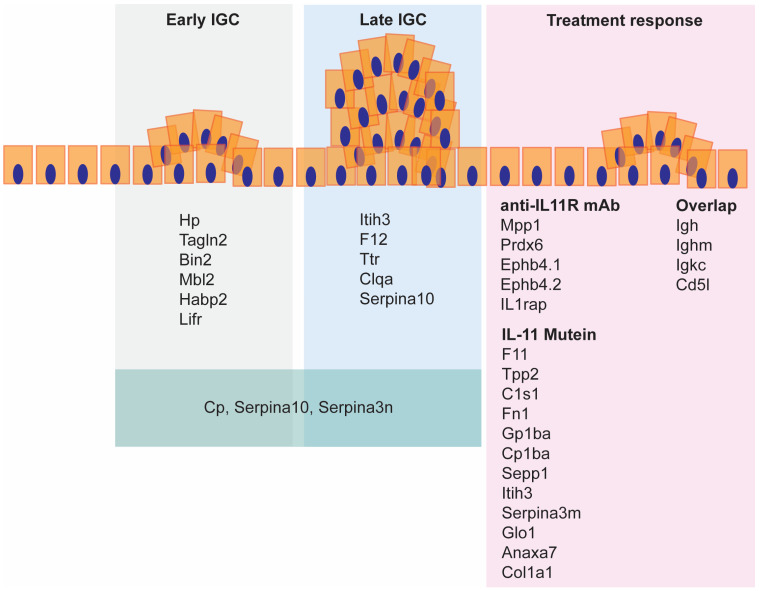
Summary of the serum proteome changes observed during IGC progression and tumor regression.

We identified serum biomarkers that were exclusive to early or late IGC (Figure 5). Each of the markers we identified have previously been linked to human cancer, and thus are not limited in relevance to the murine model employed. Of these, Tagln2 has previously been linked to angiogenesis, proliferation, migration, and epithelial–mesenchymal transition in GC [25,26], while Mbl2 gene variants have been linked to risk of stomach cancer [27] and increased serum levels linked to pancreatic cancer [28]. In addition, Itih3 has previously been identified as a predictor of GC in mice and patients [29], while F12 is a prognostic marker for thyroid cancer [30]. Previous studies have shown that Serpina3n levels correlate with inflammation in colon cancer [31], and promotes invasion and migration in breast cancer [32].

### 3.2. Comparison of Two Treatment Modalities That Block IL-11 Signaling

IL-11 has been linked to the onset and progression of numerous solid malignancies and has been gaining increased interest as a novel therapeutic target [33]. In GC, IL-11 is associated with tumor progression, invasion, and poor survival outcomes [34,35,36,37]. Thus, utilization of the *gp130*^Y757F^ model of GC, in which IL-11 has a major role in GC formation [15], allowed for a unique opportunity to explore whether markers of tumor regression following treatment could be detected within the serum proteome.

To provide future directions to improve IGC patient selection and treatment outcome, we compared two different inhibitors of IL-11 signaling, namely a monoclonal antibody and a recombinant protein. While both inhibitors showed similar therapeutic efficacy, IL-11 Mutein, the recombinant protein revealed different markers of treatment response (Figure 5). Interestingly, our results revealed that changes in proteins associated with the tumor matrix were present following both treatment modalities and may thus represent an opportunity for the development of companion diagnostics to track tumor regression.

### 3.3. Serum Biomarkers of Tumor Regression

We found changes in numerous proteins at the end of the anti-IL-11R treatment compared to controls (Figure 5). Of these, Mpp1 is associated with the cytoskeleton and cell proliferation [38], Prdx6 is associated with migration and invasion in colon cancer [39], and Epb4.2 predicts survival outcomes in pancreatic cancer [40]. It has also been shown that il1rap regulates apoptosis in the stomach in GC models [41]. Ephb4.1 and Ephb4.2 are frequently elevated in tumors [42] and may be a reflection of residual disease. At the end of the IL-11 Mutein treatment, we observed changes in proteins such as Tpp2, which is implicated in cellular division and apoptosis and is significantly increased in squamous cell carcinomas [43], while C1s1 and Gp1ba have been linked to fibroblasts and matrix in other solid tumor types [44,45], suggesting matrix remodeling may occur following treatment.

We also compared serial serum samples from the same mouse. While we did not detect changes in response to the anti-IL-11R treatment until the end of the treatment, we did detect changes in response to the IL-11 Mutein treatment throughout the treatment cycle. This included changes in Glo1 and Anxa7 which have previously been shown to be elevated in GC tumors [46]. Col1a1 is overexpressed in GC [47], and its reduction following IL-11 Mutein treatment further suggests remodeling of the tumor matrix.

### 3.4. Strengths and Limitations

By comparing healthy animals to treatment-naïve *gp130*^Y757F^ animals with early- and late-stage GC, we were able to identify proteins that were unique to each disease stage and eliminate proteins that may have been associated with aging rather than the presence of a cancer.

We used serial samples from the same animals which allowed for robust tracking of treatment success between the two modalities. Our previous studies have shown that IL-11 Mutein is efficacious in this GC model [15]. Here, we demonstrate that a monoclonal antibody against IL-11R had comparable efficacy and safety to IL-11 Mutein-mediated inhibition of signaling, and thus enabled a head-to-head comparison of changes in the serum proteome throughout treatment between the different modalities.

## 4. Materials and Methods

### 4.1. Mice

All animal experiments were conducted in accordance with the Animal Ethics Committee (AEC) of the Ludwig Institute for Cancer Research (AEC11/07 approved 2011) and the Walter and Eliza Hall Institute, Australia (AEC 2012.019 approved 2012). Age- and gender-matched *gp130*^Y757F^ mice or wild-type mice on a mixed 129/C57BL/6 background [11] were maintained in a specific pathogen-free barrier facility.

For therapeutic studies, 6.5–9.5-week-old gender-matched *gp130*^Y757F^ mice received intraperitoneal injections of 50 mg/kg recombinant human IL-11 Mutein-PEG [15] three times/week or 15 mg/kg of a murine specific anti-IL-11R monoclonal antibody (CSL-4E5 mG1k) [20] once/week over 4 weeks. Control animals received 15 mg/kg of an isotype control (CSL-3M4 mG1k). Sub-mandible bleeds were performed before the first treatment and at the end of the second week of treatment. Terminal cardiac bleeds were performed at the end of the treatment protocol. Serum was collected in microvette 500 Z-gel serum tubes and stored at −80 °C.

On autopsy, stomachs were excised, cut along the greater curvature and pinned with the lumen open. Macroscopic gastric tumors were enumerated according to their size and wet mass and representative polyps and adjacent (non-cancerous) antral tissue was prepared for histological analysis. Comparisons between values from two groups were performed using Student’s *t*-test (two tailed). * *p* < 0.05. ** *p* < 0.01. *** *p* < 0.001.

### 4.2. Gene Expression

For isolation of gastric cell populations, tissue was digested in 5 mM EDTA, RNA was extracted using TRIzol reagent (Invitrogen, Waltham, MA, USA), and cDNA was prepared using a High Fidelity cDNA synthesis kit (Applied Biosystems, Waltham, MA, USA) as per the manufacturer’s instructions. Quantitative RT-PCR gene expression analysis was performed in triplicate using taqman probes (Applied Biosystems) on an ABI7300. Expression data were normalized to *Gapdh* mRNA expression.

### 4.3. Proteomics

#### 4.3.1. Immunoaffinity Chromatography

High-abundance proteins were depleted from the *gp130*^Y757^ mouse serum using the 4.6 × 50 mm Multiple Affinity Removal System^™^ (MARS) column MS-3 (Agilent Technologies, Santa Clara, CA, USA) which specifically removes albumin, IgG, and transferrin. Depletion was performed at room temperature with an Agilent 1100 series HPLC system as per manufacturer’s instructions. Serum samples were diluted four-fold in MARS buffer A and centrifuged at 13,000× *g* for 5 min to remove particulates. Low-abundance protein fractions (representing depleted serum) were collected and stored at −20 °C.

#### 4.3.2. Proteomics Sample Preparation

Serum obtained from mice within the therapeutic studies were diluted four-fold in Tris/Urea buffer (0.1 M Tris-HCl, 8 M urea, pH 8.5) and 5 μL diluted serum from each mouse was prepared for mass spectrometry analysis using the FASP digestion method as previously described [48]. Depleted serum fractions (400 μL) from *gp130*^Y757^ mice were also subjected to FASP digestion.

#### 4.3.3. High pH Peptide Fractionation

Tryptic peptides (3 μL) from each of the *gp130*^Y757^ mice were pooled across the samples and subjected to high pH reversed-phase analysis on an Agilent 1100 Series HPLC system equipped with a variable wavelength detector (280 nm). Fractionation was performed on XBridge™ Shield C_18_ column (10 × 100 mm, 3.5 μm bead size, Waters, Milford, MA, USA). Peptides were separated by their hydrophobicity at a high pH at a flow rate of 0.1 mL/min using a gradient of mobile phase A (5 mM ammonium formate, pH 10) and a mobile phase B (100% acetonitrile, ACN), from 3% to 45% over 70 min. Fractions were collected every minute across the gradient length and concatenated into 27 fractions. Eluted peptides were dried in a SpeedVac centrifuge (CentriVap, Labconco, Kansas City, MO, USA) and reconstituted in MS loading buffer (2% ACN/0.1% FA) prior to MS analysis.

#### 4.3.4. Mass Spectrometry Experimental Design

Peptide mixtures (1 μL of samples, 2 μL for fractions) were separated by reverse-phase chromatography on a C18 fused silica column (I.D. 75 µm, O.D. 360 μm × 25 cm length) packed into an emitter tip (IonOpticks, Melbourne, VIC, Australia), using a nano-flow HPLC (M-class, Waters). The HPLC was coupled to an Impact II UHR-QqTOF mass spectrometer (Bruker, Bremen, Germany) using a CaptiveSpray source and nanoBooster at 0.20 Bar using acetonitrile. Peptides were loaded directly onto the column at a constant flow rate of 400 nL/min with 0.1% formic acid in MilliQ water and eluted with a 90 min linear gradient from 2 to 34% buffer B (99.9% acetonitrile and 0.1% formic acid). Mass spectra were acquired in a data-dependent manner including an automatic switch between MS and MS/MS scans using a 1.5 s duty cycle and 4 Hz MS1 spectra rate, followed by MS/MS scans at 8–20 Hz dependent on precursor intensity for the remainder of the cycle. MS spectra were acquired between a mass range of 200–2000 *m*/*z*. Peptide fragmentation was performed using collision-induced dissociation. Raw files consisting of high-resolution MS/MS spectra from the Bruker Impact II instrument were processed with MaxQuant (version 1.5.8.30) for feature detection and protein identification using the Andromeda search engine [49,50]. Extracted peak lists were searched against the Mus musculus (UniProt, October 2016) databases as well as a separate reverse decoy database to empirically assess the false discovery rate (FDR) using strict trypsin specificity allowing up to two missed cleavages. In the main search, precursor mass tolerance was 0.006 Da and fragment mass tolerance was 40 ppm. The minimum required peptide length was set to seven amino acids. Modifications: Carbamidomethylation of Cys was set as a fixed modification, while N-acetylation of proteins and oxidation of Met were set as variable modifications. LFQ quantification was selected, with a minimum ratio count of 2. PSM and protein identifications were filtered using a target-decoy approach at an FDR of 1%. The mass spectrometry proteomics data have been deposited to the ProteomeXchange Consortium via the PRIDE [51] partner repository with the dataset identifier PXD043224.

#### 4.3.5. Data Processing and Statistical Analysis

Data processing and analysis were performed using R (version 4.1.0). The false hits, including contaminants, reverse proteins, and proteins identified by site were removed. Only proteins that were quantified in at least 50% of replicates in at least one condition were kept. The protein intensities were log_2_-transformed. Missing values were imputed by using Missing Not At Random (MNAR) method. This was achieved by substituting ‘NAs’ with numbers that were drawn from a normal distribution with a mean that was left-shifted from the sample mean by 1.8 standard deviation with a width of 0.3 [52].

#### 4.3.6. Differential Expression and Enrichment Analysis

Data were normalized using RUVIIIC [53]. The optimum k value used to remove the unwanted variation was determined based on PCA, RLE, and *p*-value distribution plots. The R-package limma (v. 3.48.3) was used to perform the differential analysis [54]. A protein was determined to be significantly differentially expressed if the false discovery rate (FDR) was ≤0.05. R-packages ggplot2 (v. 3.3.5) and pheatmap (v. 1.0.12) were used to visualize the results. GO enrichment analysis of the significant gene set was performed using clusterProfiler (v.4.1.3) R-package. A GO term was determined to be significant if the false discovery was ≤0.05.

### 4.4. Conclusions and Future Prospects

Taken together, our results highlight the potential to identify reliable biomarkers to track IGC tumor regression in response to treatment. Future studies utilizing large biobanks of human IGC samples, taking into account genetic ancestry, *Helicobacter pylori* status, and other clinical risk factors, will enable validation of the biomarkers that we have identified and inform selection of the most robust marker to be developed as potential diagnostic.

## Data Availability

Data is contained within the article and Supplementary Materials.

## Supplementary Materials

The following supporting information can be downloaded at: https://www.mdpi.com/article/10.3390/ijms25063129/s1.
