# Supplementary material for: Identification of Serum Biomarkers to Monitor Therapeutic Response in Intestinal-Type Gastric Cancer
Source: Int J Mol Sci. 2024 Mar 8;25(6):3129. doi: 10.3390/ijms25063129 (PMC10970471; doi:10.3390/ijms25063129)
Supplement: Supplementary file 1 [file ijms-25-03129-s001.zip › ijms-2865203-supplementary.pdf]

Supplemental Information

Identification of serum biomarkers to monitor therapeutic response in intestinal-type gastric cancer

Laura F. Dagley [1,2], Jumana Yousef [1,2], Adele Preaudet [1,2], Andrea Loving [4], Andrew I. Webb [1,2], Matthias Ernst [5,6#] and Tracy L. Putoczki [1,2,3,#*]

[1] The Walter and Eliza Hall Institute of Medical Research, Victoria, Australia 3052
[2] Department of Medical Biology, The University of Melbourne, Victoria, Australia 3052
[3] Department of Surgery, The University of Melbourne, Victoria, Australia 3052
[4] Ludwig Institute of Cancer Research, Victoria, Australia 3052
[5] The Olivia Newton John Cancer Research Institute, Victoria, Australia 3084
[6] School of Cancer Medicine, La Trobe University, Victoria, Australia 3084

#Equal contribution
*Correspondence: Putoczki.t@wehi.edu.au; Tel.: +61-3-9345-2387

Supplemental Figures

Supplemental Figure S1 relates to Figure 1

Supplemental Figure S2 relates to Figure 3

Supplemental Figure S3 relates to Figure 3

Supplemental Figure S4 relates to Figure 4

Supplemental Figure S5 relates to Figure 4

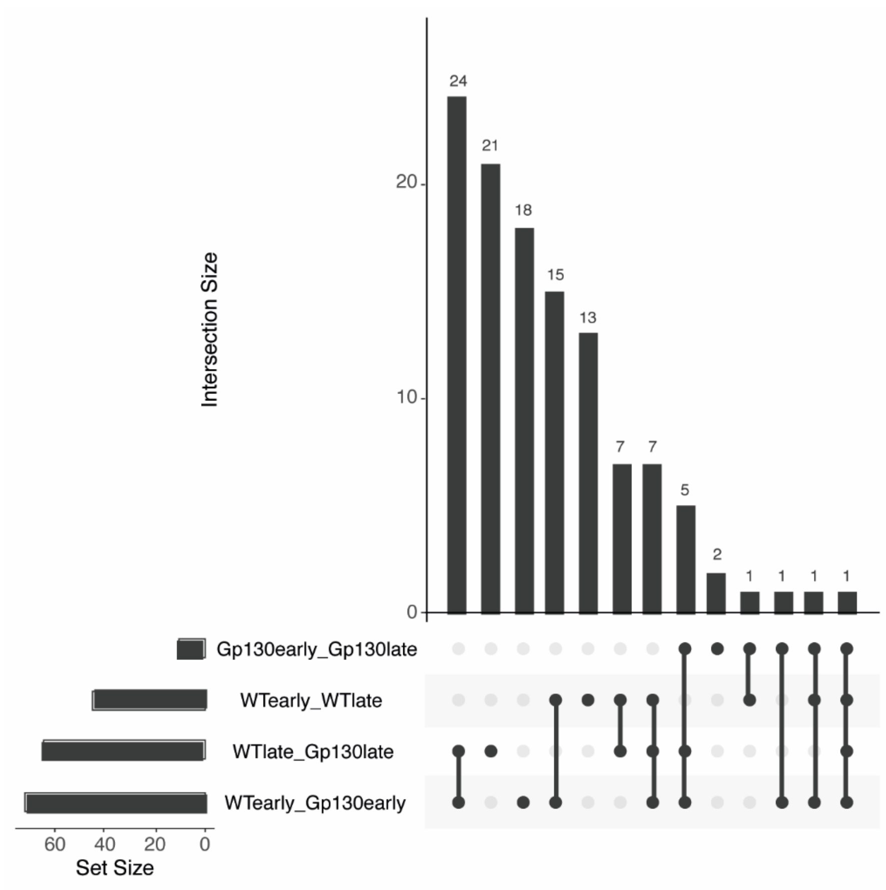

Figure S1. UpSetR plot summarizing differential protein expression analysis across the four pairwise analyses across early and late stage GC. The horizontal bar graph at the bottom left shows the total number of proteins with significant differences in $\log_2$ fold change for each pairwise analysis. Joined black circles to the right of these bar graphs indicate that the same differentially expressed proteins were common to the pairwise comparisons shown at left. The vertical bar graph at the top quantitates the number of unique proteins with significant $\log_2$ fold change expression differences in each of the pairwise comparisons.

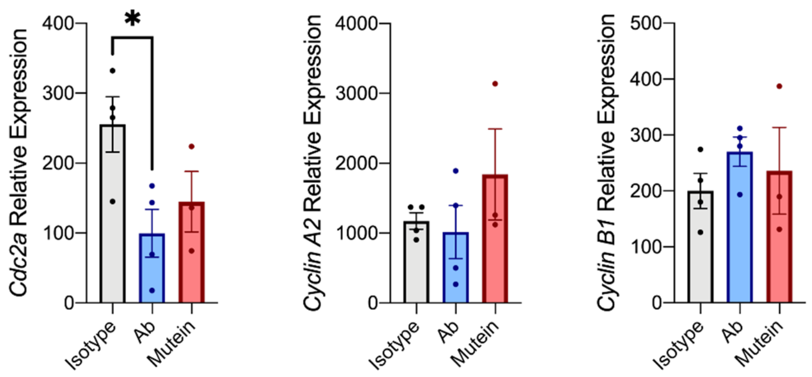

Figure S2. Inhibition of IL-11 signalling alters epithelial proliferation. Relative gene expression for isolated gastric epithelial cells from each treatment group presented +/- SEM. (**a**) *cdc2a*, (**b**) *cyclin A2*, **(c)** *cyclin B1*. * $p<0.05$ Student's T-test.

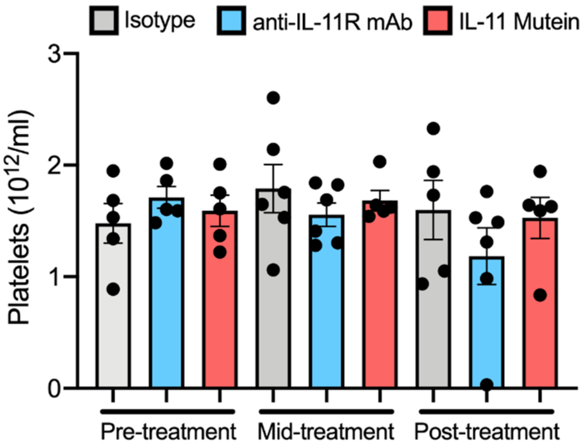

Figure S3. Platelet counts do not change following treatment. *gp130*^{Y757F} mice with established gastric tumors were treated with an isotype control, anti-IL11R antibody, or IL-11 Mutein for 4 consecutive weeks. Blood was collected before the start of the treatment regime, at the end of the second week, and at termination of the experiment.

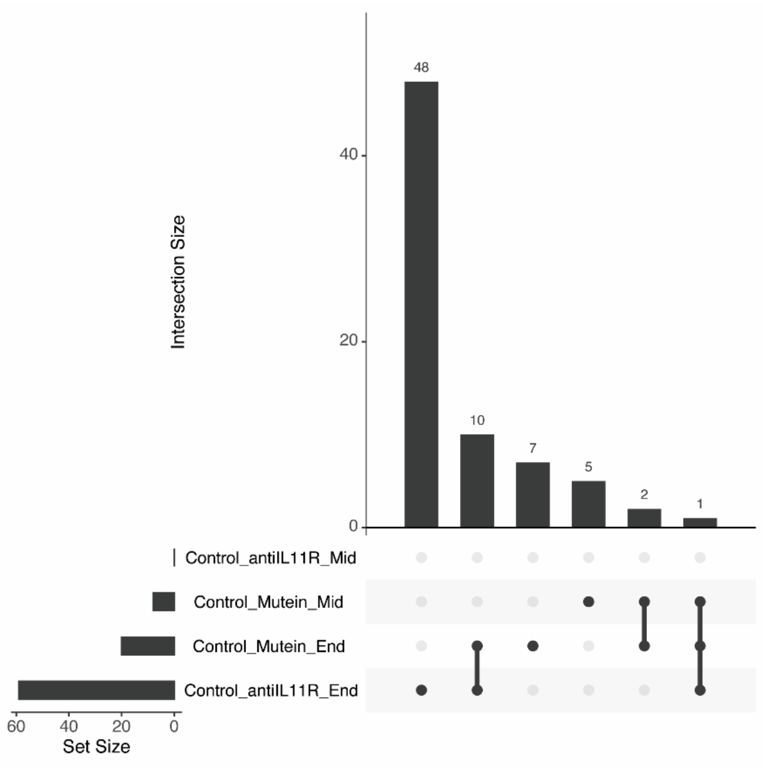

Figure S4. UpSetR plot summarizing differential protein expression analysis across the four pairwise analyses following therapeutic inhibition of IL-11 signaling. We are comparing pre-treatment (Pre) versus part way through the treatment protocol (Mid) or compared to the end of treatment (End) for both the anti-IL-11R mAb treatment or Mutein treatment as compared with the control treatment . The horizontal bar graph at the bottom left shows the total number of proteins with significant differences in $\log_2$ fold change for each pairwise analysis. Joined black circles to the right of these bar graphs indicate that the same differentially expressed proteins were common to the pairwise comparisons shown at left. The vertical bar graph at the top quantitates the number of unique proteins with significant $\log_2$ fold change expression differences in each of the pairwise comparisons.

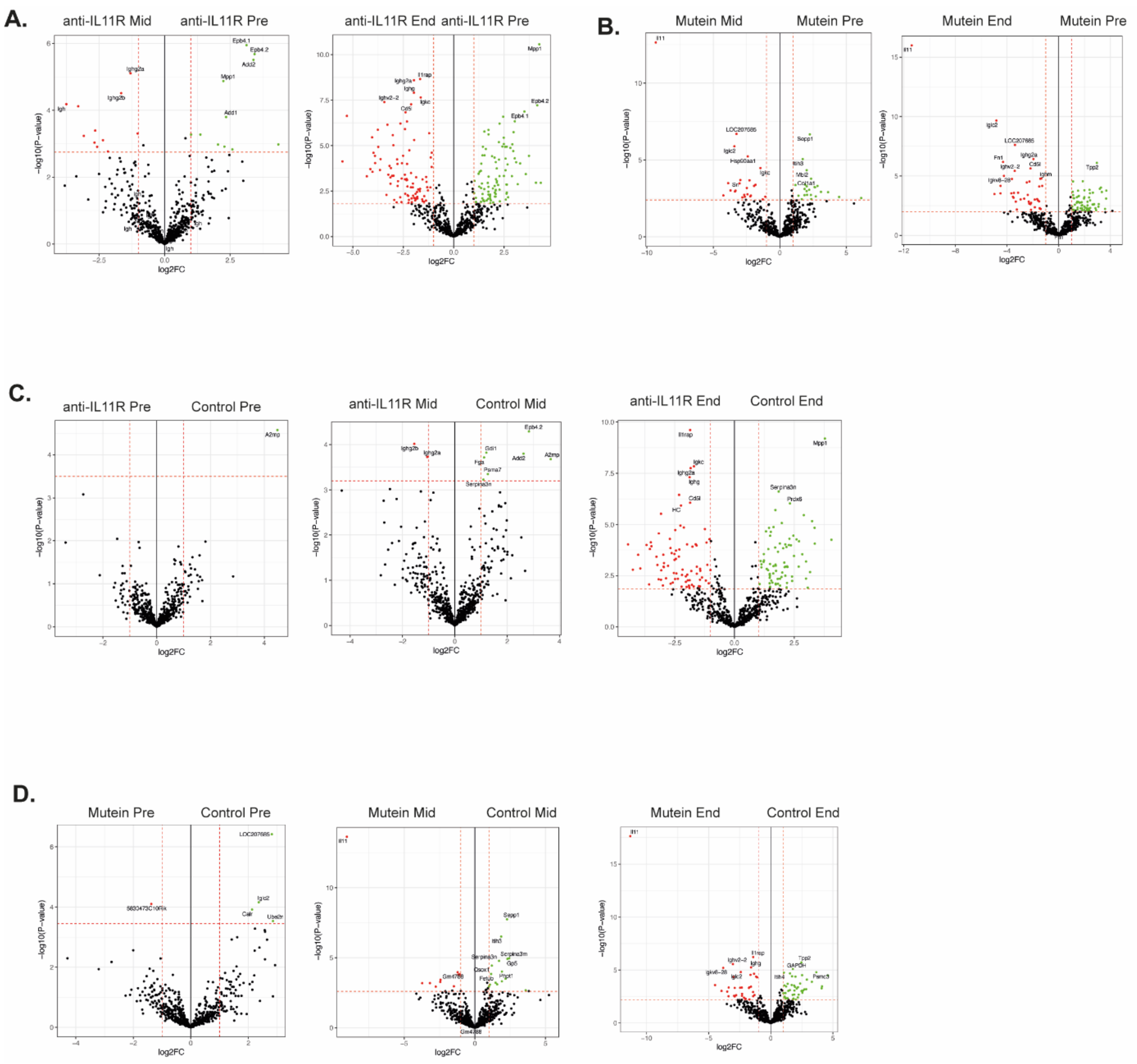

Figure S5. Volcano plots illustrating the $\log_2$ protein ratios comparing pre-treatment vs mid-treatment, and pre-treatment vs end treatment for **(A)** anti-IL-11R antibody and **(B)** IL-11 Mutein. In addition, untreated controls compared to pre, med and end treatments for **(C)** anti-IL-11R antibody and **(D)** IL-11 Mutein. Proteins were deemed differentially regulated in the log2 fold change in protein expression was $\geq$ 1-fold and exhibited an adjusted *p*-value ≤ 0.05.
